# Statins Induce a DAF-16/Foxo-dependent Longevity Phenotype via JNK-1 through Mevalonate Depletion in *C. elegans*

**DOI:** 10.14336/AD.2019.0416

**Published:** 2020-02-01

**Authors:** Andreas Jahn, Bo Scherer, Gerhard Fritz, Sebastian Honnen

**Affiliations:** Heinrich Heine University Dusseldorf, Medical Faculty, Institute of Toxicology, D-40225 Dusseldorf, Germany

**Keywords:** mevalonate pathway, statins, *C. elegans*, insulin/IGF-1 like signaling, lifespan

## Abstract

Statins belong to the most pre-scribed cholesterol lowering drugs in western countries. Their competitive inhibition of the HMG-CoA reductase causes a reduction in the mevalonate pool, resulting in reduced cholesterol biosynthesis, impaired protein prenylation and glycosylation. Recently, a cohort study showed a decreased mortality rate in humans between age 78-90 going along with statin therapy, which is independent of blood cholesterol levels. As *C. elegans* harbors the mevalonate pathway, but is cholesterol-auxotroph, it is particularly suitable to study cholesterol-independent effects of statins on aging-associated phenotypes. Here, we show that low doses of lovastatin or a mild HMG-CoA reductase knockdown via *hmgr-1(RNAi)* in *C. elegans* substantially attenuate aging pigment accumulation, which is a well-established surrogate marker for biological age. Consistently, for two statins we found dosages, which prolonged the lifespan of *C. elegans*. Together with an observed reduced fertility, slower developmental timing and thermal stress resistance this complex of outcomes point to the involvement of DAF-16/hFOXO3a, the master regulator of stress resistance and longevity. Accordingly, prolonged low-dose statin exposure leads to an increased expression of *jnk-1*, a known activator of DAF-16. Moreover, the beneficial effects of statins on aging pigments and lifespan depend on DAF-16 and JNK-1, as shown in epistasis analyses. These effects can be reverted by mevalonate supplementation. In conclusion, we describe a lifespan extension in *C. elegans*, which is conferred via two well-conserved stress-related factors (JNK-1, DAF-16) and results from mevalonate depletion.

Aging is the progressive, degenerative change in tissue organization and function leading to a higher probability to die [1]. One of the contributing degenerative processes is atherosclerosis, which is responsible for the majority of cardiovascular diseases (CVD), the leading cause of death worldwide [2]. The most common perpetrators of CVD are high cholesterol (hypercholesterolemia), high blood pressure, and cigarette smoking. Hypercholesterolemia has been treated with statins since 1987 [3]. The anti-hypercholesterolemia activity was first described by Endo and colleagues [4]. Today, statins are among the most frequently prescribed drugs worldwide [5]. Adhering to the right treatment scheme, statins are considered to be well-tolerated in humans. The rare side effects include muscle weakness (myopathy), muscle fiber decay (rhabdomyolysis) and liver toxicity [6]. Statins function as 3-hydroxy-3-methylglutaryl coenzyme A (HMG-CoA) reductase inhibitors. HMG-CoA reductase is the rate-limiting enzyme of the mevalonate pathway, which converts acetyl-CoA to mevalonate. In humans, mevalonate is a cholesterol precursor. Its depletion leads to an intrinsic cholesterol shortage and, one consequence is, a higher cholesterol uptake from the blood via LDL-receptor-mediated endocytosis. Ultimately this leads to decreased blood cholesterol levels [7]. The cardio-vascular protective effects of statins are usually attributed to lowered cholesterol levels. However, some studies indicate that cardio-protection by statins is at least partially or even entirely cholesterol-independent [8-12].

The cholesterol-independent effects of statins are, similar to the cholesterol-dependent ones, a result of HMG-CoA reductase inhibition and, hence, governed by mevalonate levels [7]. Cholesterol-independent effects of statins include effects on protein synthesis, mitochondrial function, protein glycosylation and protein localization [13]. Thus the synthesis of mevalonate indirectly influences a multitude of processes such as intracellular communication, cell growth, cell division, gene expression and cytoskeleton assembly [14]. Jacobs et al. (2013) showed that statin treatment of patients between the age of 70 and 90 reduces mortality, independent of blood cholesterol levels. Yet, the underlying molecular mechanism of this cholesterol-independent extension of human lifespan by statin intake is not completely understood.

In this study, we aimed to investigate effects of low-dosed statins in the model organism *C. elegans* with a special focus on stress-resistance and aging. *C. elegans* is a preferential model to elucidate drug effects on lifespan on a whole organism and on population level [15]. It is especially important that, although the mevalonate biosynthetic pathway is evolutionary well-conserved up to humans, the side branch within the metabolic pathway leading to cholesterol is missing in *C. elegans*. Being a cholesterol auxotroph organism,* C. elegans* is exceptionally suitable to study cholesterol-independent effects of statins. Here, we report that specific low-doses of lovastatin as well as genetic inhibition of the HMG-CoA reductase attenuate aging pigment accumulation. Furthermore, we determined dosages for two statins, which prolong the lifespan of *C. elegans.* Analyzing gene expression after lovastatin treatment we found that it induces *jnk-1* expression, a known activator of DAF-16/hFOXO3a. Lovastatin fails to influence aging pigment accumulation and lifespan in *jnk-1* or *daf-16* mutants and this lifespan extension is lost when mevalonate is supplemented. These observations led us to hypothesize that statins promote a DAF-16-dependent longevity phenotype via JNK-1 through HMG-CoA reductase inhibition in *C. elegans*.

## MATERIALS AND METHODS

### Chemical

Solvents used to prepare stock solutions are as follows: Lovastatin (Sigma-Aldrich: PHR1285) and simvastatin (Sigma-Aldrich: S6196) were dissolved in DMSO. We excluded DMSO effects for our *read-outs* by adjusting the solvent concentration in all experimental groups to the respective concentration of the highest treatment group as DMSO has an influence on relevant parameters for our study itself [16]. In all experiments DMSO did not exceed 0.2 %. 5-Fluoro-2′-deoxyuridine-stock (Sigma-Aldrich: F0503) was dissolved in water.

### Strains

Maintenance and handling of *C. elegans* were carried out as described previously [17]. Bristol N2 was used as the wild type strain and for backcrossing of mutant strains. Single mutant strains were as follows: VC8 [*jnk-1(gk7)*], CB1370 [*daf-2(e1370)*], CF1038 [*daf-16(mu86)*]. Transgenic strains were as follows: TJ356 [*daf-16p::daf-16a/b::GFP + rol-6(su1006)*], SJH1 [*daf-16p::daf-16a/b::GFP + rol-6(su1006), jnk-1(gk7)*] (Crossbreed of TJ356 with VC8).

### Fertility and Development

Total progeny was determined over five days starting with L4-individuals randomly distributed to permanent treatment with three lovastatin concentrations (25, 50 and 100 µM) or 0.2 % DMSO in liquid media with heat-inactivated bacteria. Every day each adult nematode was transferred to fresh media. For determination of development semi-synchronized individuals were treated with lovastatin (25, 50 and 100 µM) or DMSO in liquid media from hatching on. After 72 h developmental stages within the population were determined.

### RNA-mediated interference (RNAi)

RNAi by “feeding” was performed essentially as described before [18]. The RNAi feeding clones were obtained from the Ahringer RNAi library (Geneservice Limited, UK). Clones were verified by sequencing. HT115(DE3) bacteria carrying the empty L4440 vector were used as controls.

### Lifespan assay

Lifespan assays were conducted in 12-well plates at 20°C in liquid medium including heat-inactivated OP50 (*E. coli*) on a rotator plate (50 rpm) in the dark or on solid media (NGM-agar) plates seeded with heat-inactivated OP50 (*E. coli*). L4/young adult animals were incubated with the respective drug or solvent (DMSO). DMSO did not exceed the final concentration of 0.2 % and was equal in all experimental groups. For RNAi treatments solid media (NGM-agar) plates containing IPTG and ampicillin seeded with HT115 (*E. coli*) were used. Within the reproductive phase the animals were transferred every other day, later only when needed. 5-Fluoro-2′-deoxyuridine (final concentration 25 µM) was added at days five to eight (72 h). Every weekday the live-dead determination was made by touch provoked movement. For the post-reproductive lifespan analysis day-eight adults were used.

### Aging pigment accumulation

Culture conditions and maintenance were the same as described for the lifespan assay. On days six, ten and twelve, the accumulation of the aging pigments was evaluated by fluorescence microscopy (extinction: 360-370 nm and emission: 420-460 nm, Olympus BX43 equipped with XM10 camera). Measurement of fluorescence intensity of the aging pigments in the intestine and the background correction were done with ImageJ.

### Stress resistance assays

Resistance to oxidative or thermal stress was determined by measuring the survival of animals after applying the respective noxe. Culture conditions were the same as described for the lifespan assay. After 24 h pretreatment nematodes were either incubated for 4 h with 2, 4 or 6 mM H_2_O_2_ or for 3, 4, 5 or 6 h at 37°C. After the respective treatment time, the survival of the individuals was evaluated by touch-provoked movement.

### DAF-16::GFP translocation

L4/young adult individuals of strains TJ356 and SJH1 were pretreated 24 h with lovastatin or the respective solvent control (0.2 % DMSO). After this, localization of DAF-16::GFP was determined for each individual by fluorescence microscopy and assigned to the three stages nuclear, intermediate or cytosolic (supplementary Fig. 1). As a positive control nematode were incubated at 37°C for 20 minutes.

### Real-time quantitative PCR (RT-qPCR) and data analysis

For RNA isolation, approximately 2000 L4 larvae were used. RNA was extracted 24 h after treatment with the appropriate condition. The animals were either rinsed with M9 buffer from the agar plates or picked up from liquid medium with a pipette and collected in an Eppendorf tube. Then animals were washed twice with M9 buffer and lysed using Tissuelyser II (Qiagen). Afterwards RNA was extracted according to instructions of RNeasy Mini Kit (Qiagen). 2000 ng mRNA were used for reverse transcription at 37°C for 1 h. Twenty microliters of quantitative PCRs was set up in 96-well plates using the following thermocycler settings: [95°C, 10 min]; 44 x [95°C 15 s, 55°C 15 s, 72°C 17 s]; [95° C 1 min, 55°C 1 min] and melting curve 65-95°C at 1°C increment, 5 s. Gene expression was normalized to four reference genes *akt-1*, *cyb-1*, *cdc-42* and *pmp-3*, using the BIO-RAD CFX MANAGER software (BIO-RAD, Hercules, CA, USA).

### Determination of antioxidative capacity in vitro

To determine the antioxidative capacity we made use of the Trolox Equivalent Antioxidative Capacity (TEAC) Assay [19]. In this assay the amount of a blue-green radical decolorized through conversion by an antioxidant is measured spectrophotometrically. The antioxidative capacity of test substances is given in comparison to the antioxidative capacity of Trolox, a water-soluble vitamin E derivative. All substances were measured in a concentration range from 0 to 25 µM. The radical scavenging activity was measured at 734 nm (Lambda 25 UV/VIS Spectrometer, Perkin Elmer, Wellesley, MA, USA) two minutes after starting the reaction. The results for each substance where plotted as a function of concentration and absorbance. To calculate the TEAC-values the gradient of the plot for the sample was divided by the gradient of the plot for Trolox.

### Pharyngeal pumping quantification

L4/young adult individuals were pretreated 24 h with 100 µM lovastatin or the respective solvent control (0.2 % DMSO). For the experiment without preincubation, untreated 1-day adult nematodes were used. They were washed twice in M9 buffer and starved for 2 h. Measuring of pharyngeal pumping was carried out with the help of Nemametrix The ScreenChip™ System in the presence of OP50 and 100 µM lovastatin respectively 0.2 % DMSO. For each nematode pumping frequency was recorded for 2 minutes.

### Chemotaxis Assay

For Chemotaxis Assay large Petri dishes (10 cm diameter) were used. Each Petri dish was filled with 20 ml of NGM agar without NaCl, Bacto Peptone and Cholesterol. Plates were divided into four quadrants (A, B, C and D) with A-B and C-D opposing each other. In the upper center of each quadrant, 1 μl of 1 M NaN_3_ was applied. After the drop of NaN_3_ had dried, 1 μl of 100 μM lovastatin was added to the top center of Quadrant A and B and 0.2 % DMSO at C and D. Approximately 100 L4 larvae were pipetted into the middle of the plate. After 2 h, animals within a 2 cm radius of either spot were counted, and a chemotactic index (CI) was calculated as stated here: (CI) = (A+B) - (C+D)/ (A+B+C+D).

**Figure 1.**
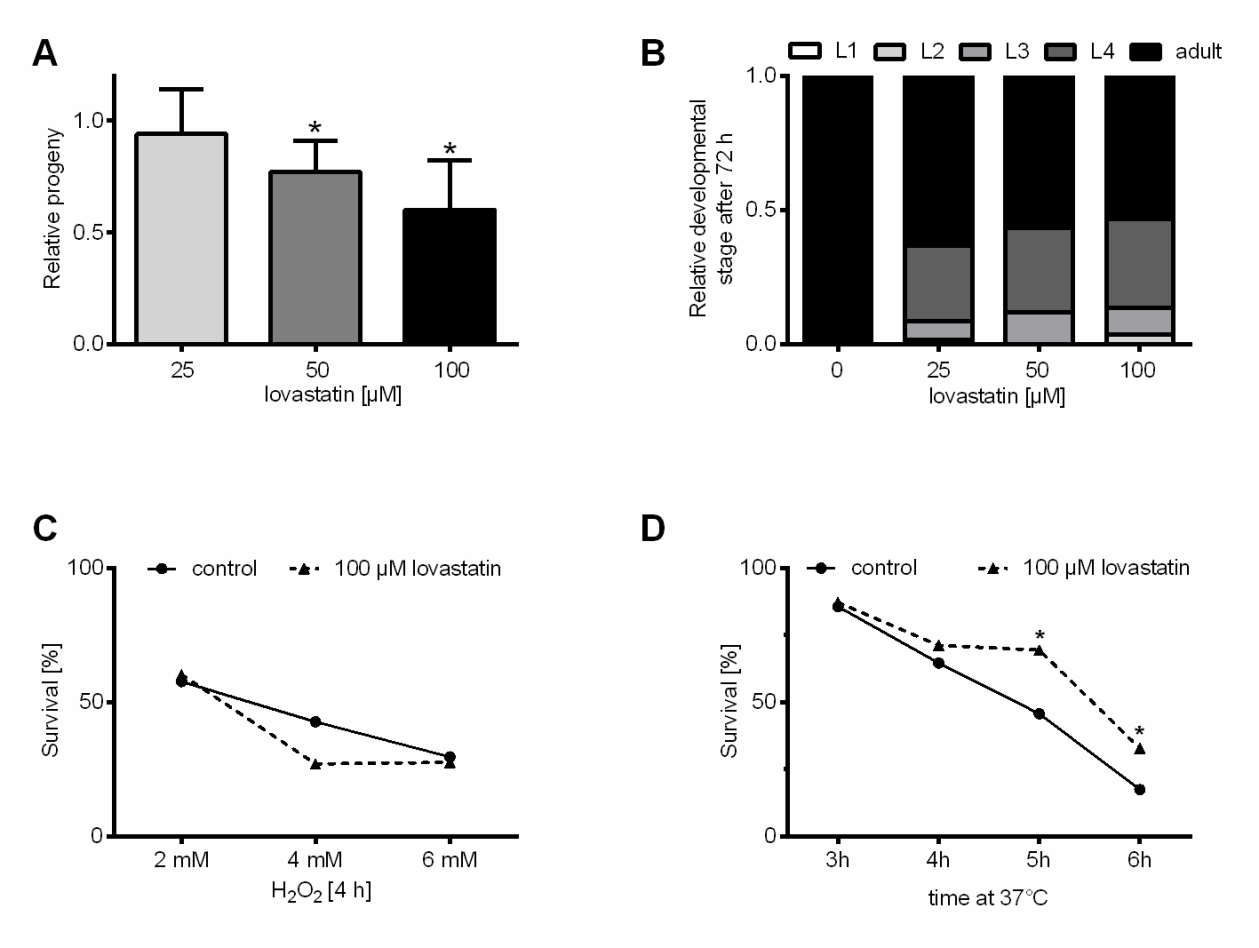
**Low dose lovastatin restrains progeny production and development but enhances thermal stress resistance in *C. elegans***. **(A)** Lovastatin treatment reduces total progeny production. Total progeny was determined over five days starting with L4-individuals randomly distributed to permanent treatment with three lovastatin concentrations (25, 50 and 100 µM) or DMSO. Shown is the percentage of progeny relative to the control population. All bars represent mean+SEM from three independent experiments, each with at least 14 individuals being analyzed (n = 3, N ≥ 42). **(B)** Continuous lovastatin treatment slightly delays development by trend. The percentage of developmental stages within lovastatin treated (25, 50 and 100 µM) and DMSO-treated control populations were determined 72 h after hatching. Bars represent mean value of three independent experiments with at least 20 individuals each (n = 3, N ≥ 60). There is a trend in the lovastatin group for a slower development with increasing statin dose. **(C)** Lovastatin does not confer resistance against H_2_O_2_-induced oxidative stress in *C. elegans*. The surviving fractions of lovastatin (100 µM, 24 h) or DMSO pre-incubated populations are indistinguishable after exposure to different H_2_O_2_ concentrations (2, 4 and 6 mM). Shown is the fraction of surviving animals in the population after four hours. Dots/triangles represent the mean from three independent experiments with at least 20 individuals each (n = 3, N ≥ 60). **(D)** Lovastatin robustly increases thermal stress resistance of *C. elegans*. After incubation of the nematodes for five or six hours at 37°C, the surviving fraction of lovastatin pre-incubated populations (100 µM, 24 h) is significantly higher as compared to DMSO-treated control population. Shown is the fraction of surviving animals in the respective population after the indicated incubation time at 37°C. Each dot/triangle represents the mean value from three independent experiments with at least 20 individuals each (n = 3, N ≥ 60). *p≤0.05 (*1way ANOVA* with uncorrected Fisher´s LSD post hoc test).

**Table 1:** Mean aging pigment accumulation in populations with different genetic backgrounds with and without statin treatment.

*Genetic background (allele) [start of treatment] group*	*Day 12: Mean aging pigments ± SEM (RFU)*	*Number of individuals (N)*	*% change vs. control*	*p-value vs. control*
***Wt [adult +1]***				
*control*	2454±223	52	20.3	0.152
*25 µM lovastatin*	1955±108	42	22.93	0.113
*50 µM lovastatin*	1891±88	45	40.11	0.038*
*100 µM lovastatin*	1469±231	42		
***Wt [adult +1]***				
*empty Vector*	4314±5	31	41.89	
*1:8 hmgr-1 RNAi*	2507±166	30	43.76	
*1:16 hmgr-1 RNAi*	2426±136	30	47.26	
*1:24 hmgr-1 RNAi*	2275±217	30	45.07	
*1:32 hmgr-1 RNAi*	2370±70	30		
***Wt [adult +1]***				
*empty Vector*	2339±305	30	11.42	0.35
*1:64 hmgr-1 RNAi*	1998±187	31	9.2	0.144
*1:128hmgr-1 RNAi*	2094±210	30		
***daf-16(mu86) [adult +1]***				
*control*	1146±201	20	11.41	0.038*
*100 µM lovastatin*	1276±165	21		
***daf-2(e1370) [adult +1]***				
*control*	2744±66	30	3.39	0.657
*100 µM lovastatin*	2837±139	30		
***jnk-1(gk7) [adult +1]***				
*control*	1867±282	31	7	0.716
*100 µM lovastatin*	1736±175	31		

The total N is always the result of at least three independent trials (n=3). Test for statistical difference was performed using ordinary 1way ANOVA with uncorrected Fisher´s LSD post hoc test

* p<0.05.

### Statistical analysis

“N” represents the total amount of individuals analyzed and is always the result of at least three independent trials (n=3) for statistical analysis. As given in the respective legends we performed ordinary 1way ANOVA followed by uncorrected Fisher´s LSD post hoc test using GraphPad Prism 6.0. This software was also used for the illustration of bar chart and Kaplan-Meier survival curves as well as calculating the unpaired Students t-test. The log rank post-hoc test was used for comparison between survival curves and performed using IBM SPSS Statistics 16.0. As given in the respective legends values are either expressed as mean ± standard error of the mean (SEM) or mean ± standard deviation. A value of p < 0.05 was considered statistically significant.

## RESULTS

### Low dose lovastatin increases thermal stress resistance in C. elegans

In the study by Jacobs and co-workers an increased survival was observed in populations experiencing long-term statin treatment, which was initially performed for lipid-lowering purpose. To model this situation in *C. elegans* we investigated the consequences of statin treatment with regard to general physiologic features like development, fertility and stress resistance. We selected lovastatin for our initial experiments. It is a monacolin from *Aspergillus terreus* and belongs to the first statins on the market. Therefore extensive follow up information is available for this statin [20]. It is a prodrug, which gets activated either non-enzymatically (i.e. by hydrolysis) or metabolically by members of the cytochrome P450 family, of which the *C. elegans* genome contains 80 genes [21]. It has to be noted that the anticipated serum concentration in patients, who typically take between 20 - 80 mg/day lovastatin for cholesterol-lowering reasons, is between 1-15 nM [22]. We used a concentration of 25 µM - 100 µM lovastatin administered in our culture medium. As the bioavailability of lovastatin is generally below 5 % and drug absorption in *C. elegans* is known to be poor, we hypothesize that the concentrations we used are in range of clinically relevant doses [23-25].

First, we determined the fertility of *C. elegans* populations under statin exposure and found a dose-dependent decrease (Fig. 1A). Embryonic lethality is not raised in the dose-range used (data not shown). To determine lovastatin effects on the developmental timing we started the treatment exceptionally at the time of hatching. All three statin concentrations had rather similar effects and only slowed down developmental timing by trend (Fig. 1B).

**Figure 2.**
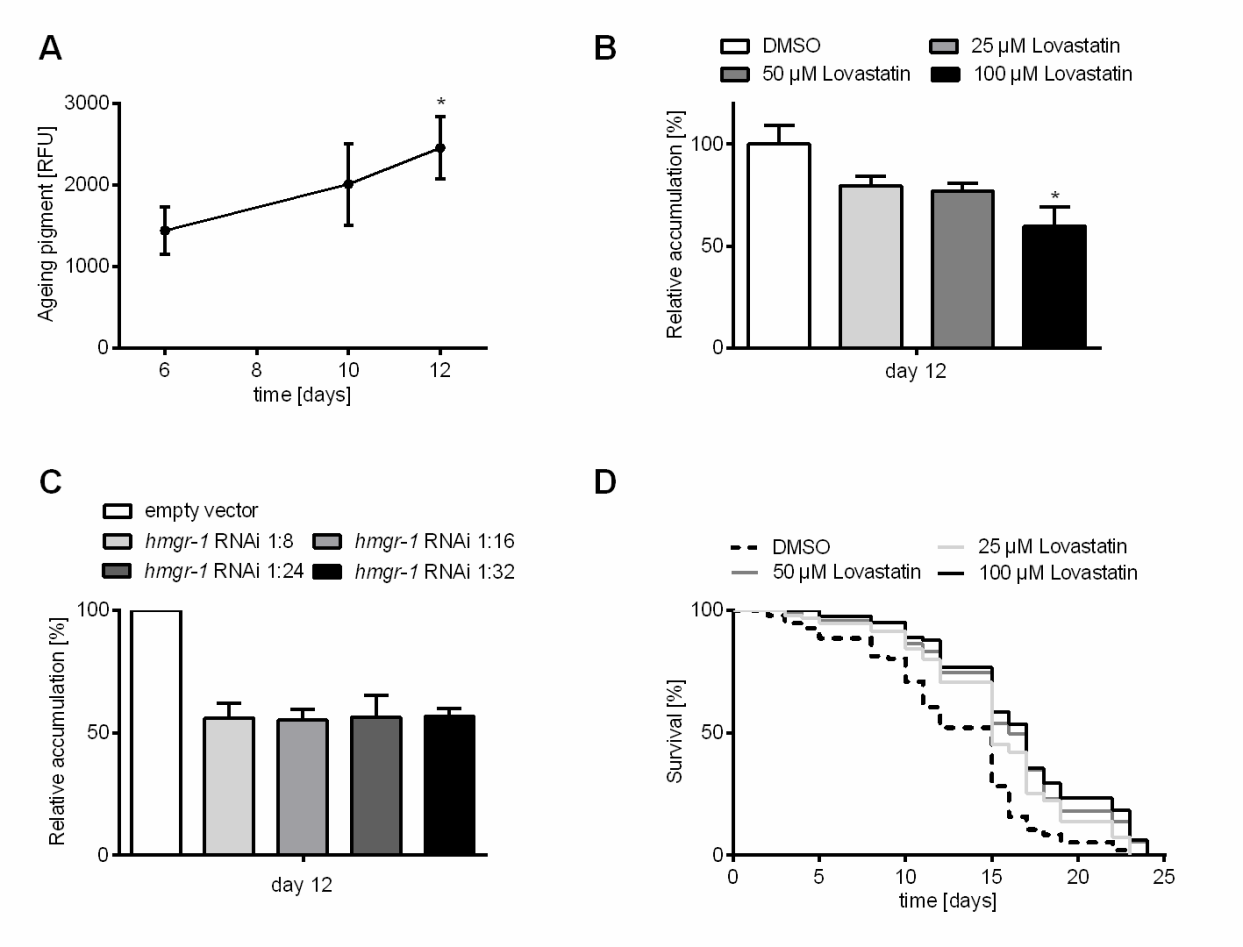
**Inhibition of HMG-CoA reductase decelerates aging pigment accumulation and extends lifespan in *C. elegans***. **(A)** Aging pigments constantly rise during aging of wild type *C. elegans*. After six, ten and twelve days of incubation with DMSO the autofluorescence was determined (DAPI filter: extinction 360-370 nm; emission 420-460 nm). Shown are the relative fluorescence units (RFU) of at least 12 individuals from three independent experiments as measured after background correction using ImageJ software for quantification (n = 3, N ≥ 36). Test for statistical difference was performed using *1way ANOVA* with uncorrected Fisher´s LSD post hoc test *p<0.05. **(B)** Low dose treatment with lovastatin robustly reduces aging pigment accumulation rate. After six, ten and twelve days of permanent treatment with different lovastatin concentrations (25, 50, 100 µM) or DMSO (vehicle control) the autofluorescence was determined (DAPI filter: extinction 360-370 nm; emission 420-460 nm) of at least 30 individuals from three independent experiments (n = 3, N ≥ 90). Expressed is the relative fluorescence normalized to the respective control. **(C)** Genetic inhibition of HMG-CoA reductase phenocopies pharmacological effects on aging pigment accumulation. After twelve days on dilutions of *hmgr-1*(RNAi) (1:8, 1:16, 1:24, 1:32) the autofluorescence was determined (DAPI filter: extinction 360-370 nm; emission 420-460 nm). Expressed is the relative fluorescence normalized to the respective control. All bars represent mean + SEM from three independent experiments with at least 10 individuals (n = 3, N ≥ 30). Test for statistical difference was performed using *1way ANOVA* with uncorrected Fisher´s LSD post hoc test *p<0.05. **(D)** Treatment with lovastatin extends the post-reproductive lifespan of *C. elegans*. Synchronized nematode populations were subdivided to DMSO (control), 25, 50 or 100 µM lovastatin on day eight of adulthood after reaching the post-reproductive stage. Shown are Kaplan-Meier survival plots for the time of treatment with lovastatin from three independent trials. The log rank post-hoc test showed statistical difference in comparison to the control for all lovastatin treatment groups (n = 3, N ≥ 80; *p≤0.05).

**Table 2:** Mean or post-reproductive lifespan of populations with different genetic backgrounds with and without statin treatment.

*Genetic background(allele) [start of treatment] group*	*Mean lifespan± SEM (days)*	*Number of individuals (N)*	*% Change vs. control*	*p-value vs. control*
***Wt [adult +1]***				
*control*	21.76±0.68	128		
*25 μM lovastatin*	24.58±0.64	132	12.96	0.004*
*50 μM lovastatin*	24.62±0.72	130	13.14	0.001*
*100 μM lovastatin*	27.10±0.76	123	24.54	<0.001*
***Wt [adult +8]***				
*control*	12.80±0.49	96		
*25 μM lovastatin*	15.22±0.48	96	18.91	<0.001*
*50 μM lovastatin*	15.94±0.50	96	24.53	<0.001*
*100 μM lovastatin*	16.63±0.51	86	29.92	<0.001*
***Wt [adult+1]***				
*control*	16.88±0.61	102		
*25 μM simvastatin*	17.30±0.56	113	2.5	0.89
*50 μM simvastatin*	19.11±0.66	107	13.2	0.001*
*100 μM simvastatin*	19.05±0.78	106	12.9	<0.001*
***daf-16(mu86) [adult +1]***				
*control*	17.11±0.33	213		
*100 μM lovastatin*	17.50±0.33	213	2.27	0.320
***daf-2(e1370) [adult +1]***				
*control*	25.13±0.73	214		
*100 μM lovastatin*	27.84±0.92	199	10.78	0.002*
***jnk-1(gk7) [adult +1]***				
*control*	22.49±0.44	186		
*100 μM lovastatin*	22.10±0.49	187	1.73	0.856

We calculated the mean lifespans within the respective observation period. Usually this is day one of adulthood (adult +1) until death, but for the post-reproductive lifespan (adult +8) it represents only the average number of days between day eight of adulthood and death. The total N is always the result of at least three independent trials (n = 3). The log rank post-hoc test was performed for determining statistical difference in comparison to the control for all treatment groups

* (p≤0.05).

Before testing general stress resistance in *C. elegans*, we measured the antioxidative capacity of lovastatin *in vitro* by using the TROLOX equivalent antioxidative capacity assay (TEAC). Lovastatin only reached a TEAC value of 0.05, indicating no antioxidative capacity *in vitro.* Many compounds such as e.g. flavonoids exert their antioxidative capacity indirectly through binding to the Antioxidative Responsive Element (ARE) or modulating other signaling pathways [26]. To test if lovastatin confers resistance against oxidative stress in *C. elegans* we performed a 24 h pre-incubation with 50, 100 and 150 µM lovastatin concentrations (supplementary Table 1) and then transferred the individuals for 4 h to hydrogen peroxide (H_2_O_2_). In this setup none of the statin pre-incubation dosages was able to influence the oxidative stress resistance of *C. elegans* as shown for the highest test dose (Fig. 1C).

Additionally, we also monitored resistance to acute thermal stress under identical pre-incubation conditions (supplementary Table 1). The different populations were transferred to high temperature liquid medium (37°C) and survival within the *C. elegans* populations was determined after three, four, five and six hours. The control population revealed a gradual decrease in survival from about 85 % after three hours over 65 % (4 h) and 45 % (5 h) to 17 % after six hours. Notably, the group pre-treated with 100 µM lovastatin revealed a 53 % larger surviving fraction than the control as observed after 5 h at 37 °C (69 %). Consistently, we detected 94 % more surviving individuals after six hours of heat stress (33 %). Hence, a 24 h pre-incubation with 100 µM lovastatin increases thermal stress resistance of *C. elegans* (Fig. 1 D).

Summarizing, our results show that short-term application of low doses of lovastatin has only mild effects on *C. elegans* fertility and development without causing lethality and, most noteworthy, improve thermal stress resistance of adult *C. elegans*.

**Figure 3.**
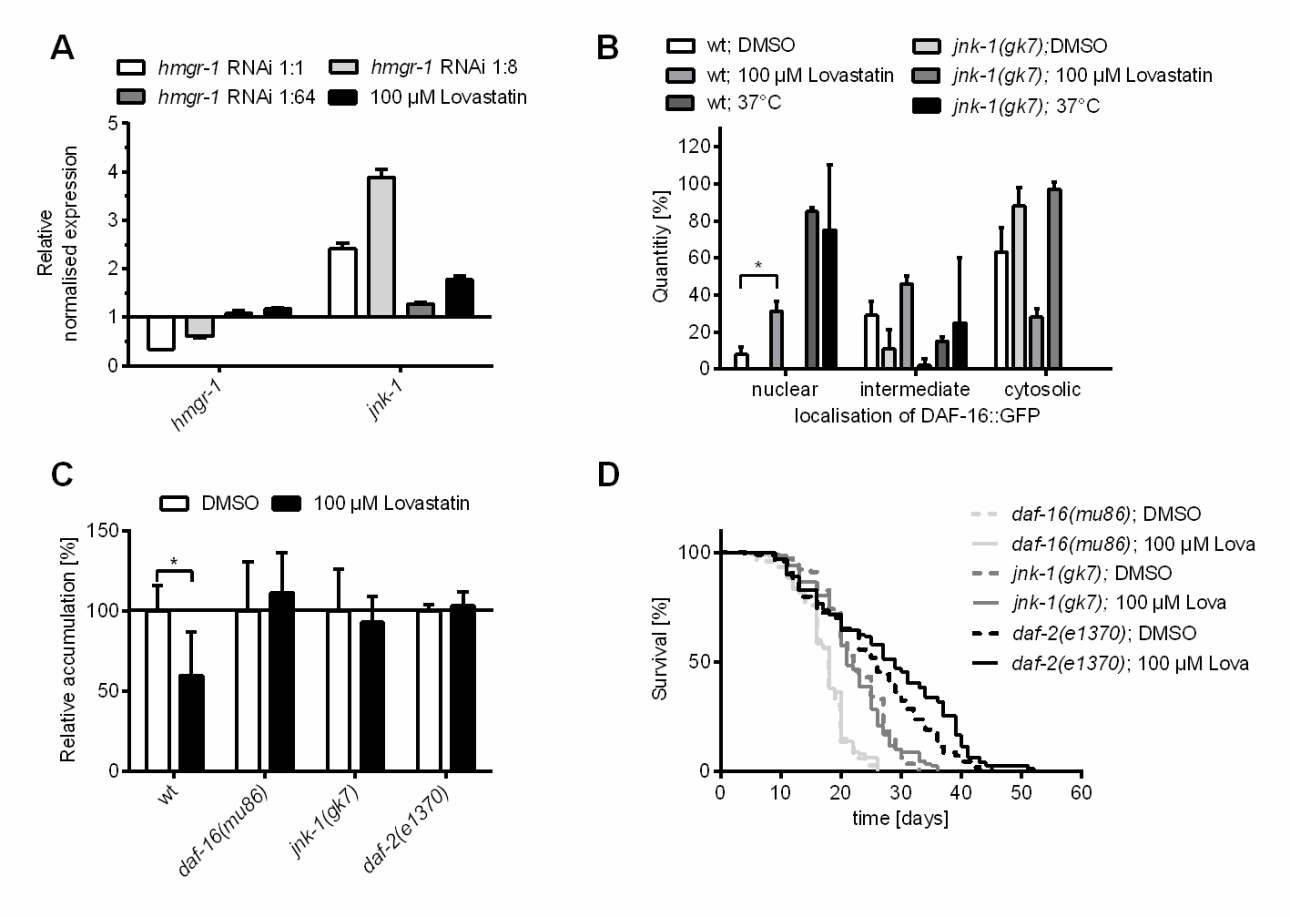
**DAF-16 is the key factor in conferring statin effects regarding aging**. **(A)** HMG-CoA inhibition enhances *jnk-1* mRNA expression. Expression levels of different candidate genes were investigated in wild-type *C. elegans* populations after 24 h treatment with: (I) vector control RNAi, (II) hmgr-1(RNAi) diluted 1:1, (III) hmgr-1(RNAi) diluted 1:8, (IV) hmgr-1(RNAi) diluted 1:64 or (V) 100 μM lovastatin. Shown are the expression changes for *hmgr-1* and *jnk-1* mRNA. *Hmgr-1* expression is reduced in case of *hmgr-1(RNAi)* in dilutions 1:1 (about 50 %) and 1:8 (about 38 %). Of interest is the fact that *jnk-1* mRNA is more abundant after *hmgr-1(RNAi)* diluted 1:8 and 100 µM lovastatin treatment, as these treatments also decrease the aging pigment accumulation. Shown is the mean ± SEM from three technical replicates (cDNA from about 2000 individuals). **(B)** Lovastatin enhances nuclear localization of DAF-16::GFP in dependence upon JNK-1. Subcellular DAF-16::GFP localization was investigated after 24 h incubation with lovastatin (25, 50, 100 µM) or 0.1 % DMSO in the wild-type (wt)- or *jnk-1(gk7)* populations. Bars represent results from three independent experiments with at least 15 individuals each (n = 3, N ≥ 45). Test for statistical difference was performed using ordinary *1way ANOVA* with uncorrected Fisher´s LSD post hoc test *p<0.05. **(C)** Lovastatin does not affect aging pigment accumulation in *daf-16, jnk-1 or daf-2* knockout populations. Wild-type-, *daf-16(mu86)-, jnk-1(gk7)* and *daf-2(e1370)* populations were continuously treated with lovastatin (100 µM) or 0.1 % DMSO. After twelve days the autofluorescence was determined (DAPI filter: extinction 360-370 nm; emission 420-460 nm). Shown is the relative fluorescence of mutants as normalized to the wild type control population (= 1.0). Bars represent the mean + SEM from three independent experiments and at least 30 individuals. Test for statistical difference was performed using ordinary *1way ANOVA* with uncorrected Fisher´s LSD post hoc test *p<0.05. **(D)** The lovastatin effect on lifespan in *C. elegans* depends on* jnk-1* and* daf-16*. Synchronized nematode populations were subdivided to 0.1 % DMSO (control) or 100 µM lovastatin on day one of adulthood. Shown are Kaplan-Meier survival plots for the time of treatment with lovastatin from three independent trials with at least 60 individuals each (n = 3, N ≥ 180. The log rank post-hoc test showed statistical difference in comparison to the control for *daf-2(e1370)* (*p≤0.05).

### Inhibition of HMG-CoA reductase decelerates aging pigment accumulation in C. elegans

After having identified doses of lovastatin, which have no appreciable adverse effects in *C. elegans* and confer thermal stress resistance we tested the accumulation of endogenous fluorescent compounds (“aging pigments”) over time. Accumulation of aging pigments is a biomarker of aging in the nematode and beyond [1]. The particles accumulating throughout the lifespan of animals are of different origin. For instance, lipofuscin is generated from oxidized and cross-linked proteins, lipids and carbohydrates or the non-enzymatic sugar addition to free amino groups of proteins leading to advanced glycation end-products (AGE). These pigments are readily visualized by fluorescence microscopy and they correlate with physiological rather than chronological age [27]. The level of aging pigments rises by about factor two between day six and twelve of adulthood, corresponding to the decline in the health status of the post-reproductive nematode (Fig. 2A). Treating animals with different concentrations of lovastatin from day one of adulthood led to a reduced accumulation of aging pigments (Fig. 2B, supplementary Fig. 2, Table 1). Treatment with 100 µM lovastatin caused a reduced accumulation of aging pigments (about 40 %) compared to the control group. To further examine if HMG-CoA reductase inhibition is responsible for the slower accumulation of aging pigments we investigated the outcome of genetic inhibition of HMG-CoA reductase using RNA interference. In line with the results for lovastatin, we observed a decline in aging pigment accumulation (about 40 %) after RNAi against the HMG-CoA reductase (Fig. 2C, supplementary Fig. 3, Table 1). It has to be noted that we had to dilute the RNAi-bacteria for this experiment as undiluted and 1:2 diluted *hmgr-1(RNAi)* led to a bloated looking intestine (data not shown) and to the animals dying early. The level of aging pigments remained the same in 1:8 - 1:32 dilutions of RNAi bacteria. Higher dilutions (1:64 or 1:128) resulted in the same levels also found for the untreated population (Table 1; supplementary Fig. 4). Therefore, we conclude that a slight genetical or pharmacological inhibition of the HMG-CoA-reductase can reduce the accumulation of aging pigments in *C. elegans.*

### Lova- and simvastatin extend the lifespan of C. elegans

As decelerated accumulation of aging pigments is often described to be indicative for a longevity-phenotype we sought to examine whether statins also confer an increased lifespan in *C. elegans*. Indeed, constant incubation with lovastatin starting from day one of adulthood prolongs lifespan of the respective *C. elegans* populations (Table 2; supplementary Table 3). 100 µM lovastatin increased the mean lifespan by about 25 % (5.5 days). As we observed a considerable reduction in fertility resulting from treatment with lovastatin, we aimed at ruling out with a potential interference with lifespan. Hence, we also investigated the effects of lovastatin treatment on the post-reproductive lifespan (Start of treatment: Day 8 of adulthood). Although the statin dose was lower in this case, as the time of treatment was reduced by eight days (roughly 25 %), the induced longevity phenotype was not weakened under these conditions (25 µM, 50 µM and 100 µM; Fig. 2D; Table 2; supplementary Table 3), with 100 µM lovastatin conferring a lifespan extension of about 30 % (4 days). Noteworthy, lifespan is undistinguishable from control conditions when mevalonate is supplemented in parallel to lovastatin. (supplementary Fig. 5). In addition, we tested simvastatin, a statin drug with higher relevance on the market today. Again, we found dosages (50 and 100 µM), which increased the mean survival time of *C. elegans* (Table 2). To rule out the possibility that dietary restriction is artificially induced by lovastatin we measured the pharyngeal pumping frequency and the chemotaxis properties of lovastatin. We revealed that neither pharyngeal pumping frequency in the presence of OP50 is reduced by lovastatin nor has lovastatin on its own a repellent effect on* C. elegans* (supplementary Fig. 6, 7 and supplementary Table 2).

### Lovastatin treatment leads to a DAF-16/FOXO-dependent longevity phenotype via JNK-1

To further elucidate the molecular mechanism of the longevity caused by HMG-CoA reductase inhibition we determined the mRNA expression levels of diverse candidate genes involved in the regulation of stress resistance and lifespan. In particular, the induction of the Jun N-terminal Kinase *(jnk-1)* through lovastatin and *hmgr-1(RNAi)* raised our attention (Fig. 3A). We also confirmed by RT-qPCR that undiluted RNAi reduced the amount of HMG-CoA reductase mRNA by more than 50 %, a 1:8 dilution only by about 38 % while 1:64 diluted *hmgr-1(RNAi)* or lovastatin had no effect on the *hmgr-1* mRNA abundance (Fig. 3A). It is known that JNK-1 is able to activate the transcription factor DAF-16, the master regulator of stress resistance and lifespan, by phosphorylation [28]. Having this in mind, we examined if exposure to lovastatin influences the localization of DAF-16 in a transgenic reporter strain ubiquitously overexpressing DAF-16::GFP. Under control conditions (20 °C, liquid medium) the transcription factor is distributed more in the cytoplasm and only partially in the nucleus. Upon treatment with lovastatin we observed a higher amount of DAF-16::GFP in the nuclei (Fig. 3B). In addition, we analyzed the expression of five DAF-16 target genes and the expression pattern after lovastatin treatment fits to a DAF-16 activation (supplementary Fig. 8). To verify that the observed statin-induced overexpression of *jnk-1* is causally related to the translocation of DAF-16 into the nucleus after HMG-CoA reductase inhibition, we crossed the *jnk-1(gk7)* deletion mutant with the respective DAF-16::GFP reporter strain. In this strain DAF-16::GFP does not translocate into the nucleus after treatment with lovastatin (Fig. 3B). These results suggest that JNK-1 is an important effector of HMG-CoA reductase inhibition, affecting both stress resistance and lifespan.

To gain further insight into the mode-of-action of this statin effect we also investigated the accumulation of aging pigments in the *daf-16(mu86)* mutant strain as well as in the *jnk-1(gk7)* and the *daf-2(e1370)* lines. DAF-2 is the Insulin/IGF-1 receptor homolog in *C. elegans* and is known as the major negative regulator of DAF-16 activity in *C. elegans*. In *daf-2* knockout strains DAF-16 is overactive *per se*. Lovastatin did not attenuate the accumulation of aging pigments in these knockout strains (Fig. 3C; supplementary Fig. 9). As JNK-1 and DAF-2 influence DAF-16 in an opposing manner, we originally hypothesized that lovastatin has no effect in the *jnk-1(gk7)* strain, whereas in *daf-2(e1370)* we anticipated to still observe some effect on aging pigment accumulation. To further clarify this discrepancy, we additionally examined the influence of lovastatin on the whole lifespan in all strains. The results of these extensive studies revealed that lovastatin further increased the already elevated lifespan of *daf-2(e1370)* animals by 11 % (2.7 days) but had no effect in *daf-16(mu86)* or *jnk-1(gk7)* knockouts (Fig. 3D; Table 2).

## DISCUSSION

In this study, we investigated effects of low-dosed statins in the model organism *C. elegans* with a special focus on stress-resistance and aging. It was motivated by the finding that the probability to die is reduced in elderly humans through cholesterol-independent mechanisms of statins. Here we report that low-dosed lovastatin treatment as well as genetic inhibition of the HMG-CoA reductase attenuate aging pigment accumulation. Lovastatin prolongs lifespan of *C. elegans* and induces expression of *jnk-1*, a known activator of DAF-16/FOXO, as well as some DAF-16 target genes related to lifespan extension. In accordance with this result, lovastatin exposure does not attenuate aging pigment accumulation or prolongs lifespan in *jnk-1* or *daf-16* knockout populations or upon parallel mevalonate supplementation. Therefore, we hypothesize that HMG-CoA reductase inhibition induces a DAF-16-dependent longevity phenotype via JNK-1 in *C. elegans*.

As all medication also statins need to be taken within a thoroughly tested therapeutic dosage, which will be fine-tuned in collaboration with the clinician (adjustment phase). While we elucidated potential beneficial effects of continuous low-dose statin treatment, previous studies used *C. elegans* to investigate the molecular consequences of high-dosed statins. In these studies, the adverse effects of statins on muscle and mitochondria functions described by clinical data, could be reproduced and further resolved in *C. elegans*, highlighting the usefulness of this model organism in statin research [29]. Moreover, Morck *et al.* showed that high doses of statins lead to embryonic lethality in *C. elegans* due to the impaired prenylation of small GTPases [29, 30]. We also observed a reduction in fertility, but no embryonic lethality, thus underlining the chosen low-dose setting was correct.

In the present study, we employed *C. elegans* to investigate the potential beneficial effects of pharmacological (lova- and simvastatin) and genetically inhibition of HMG-CoA reductase (HMGR-1) on stress resistance and aging in general. We showed that continuous treatment with lovastatin extended the life span of *C. elegans* in a JNK-1 and DAF-16 dependent manner. This lifespan extension was not observed when mevalonate was supplemented in the culture medium in parallel to lovastatin but can also be found in the presence of simvastatin.

Testing diverse mitochondrial modulators Andreux and colleagues showed that also fluvastatin increases lifespan of *C. elegans* [31]. They conclude that a decrease in respiration induced by fluvastatin has an overall beneficial effect in the nematode. Further, they report that this decrease in respiration is probably caused by inhibition of mevalonate synthesis, which in turn limits Ubiquinone (Q10). This could affect the mitochondrial respiration chain, leading to altered mitochondrial function, which was shown before to enhance lifespan and oxidative stress resistance in *C. elegans* [32]. Nevertheless, two aspects of our findings argue against mitohormesis as a result of statin treatment. First, we only observed thermal stress resistance, but no oxidative stress resistance upon lovastatin treatment. Second, Q10 is dietarily available in the *Escherichia coli* food source to a certain extend during lifespan analysis. This connection should be further resolved in future studies. Nevertheless, from the fact that another group also reported a longevity phenotype after treatment with a different statin we conclude that the observed statin effect is a general one.

Moreover, Sapir and co-workers describe findings on sumoylation of the HMG-CoA synthase (HMGS-1) regulated by the small ubiquitin-like modifier protease ULP-4 [33]. Mutants with reduced HMGS-1 activity generate less HMG-CoA, which in turn hampers the HMG-CoA reductase resulting in lower mevalonate levels comparable to statin treatment. Noteworthy, *ulp-4* mutants live about 20 % longer than wild-type animals, which is within the range of the statin effect reported here.

We showed that lovastatin has no repellent effect on *C. elegans* and does not reduce pharyngeal pumping rate in the presence of food. Additionally, Pilon and co-workers showed that the inhibition of HMG-CoA reductase does not impact the composition of *C. elegans* fat stores as measured by coherent anti-stoke raman scattering (CARS) microscopy, indicating that statins do not interfere with lipid storage and related energy balance [30]. Hence, dietary restriction is not the reason for the observed longevity in *C. elegans.*

Further, it was shown in Drosophila melanogaster, which is also cholesterol auxotroph, that simvastatin treatment extends its lifespan [34]. Yet, it is rational to assume that the longevity phenotype we explored here in detail is not related to cholesterol levels and can likewise be found in other species.

It is evident that in humans, cholesterol-independent effects like decreased platelet activation, inhibition of cardiac hypertrophy, reduction of cytokine-mediated vascular smooth muscle cell proliferation, and improvement of endothelial function are part of beneficial effects of statin treatment [10]. Still, the presence of a longevity phenotype in *Drosophila* and *C. elegans*, in which those processes are largely absent, is intriguing, arguing for mechanisms independent of the statin influence on cardiovascular function.

Our findings suggest that JNK-1 activity is increased upon of continuous lovastatin treatment. Hence, more DAF-16 will be found in the nucleus after phosphorylation by JNK-1, which in turn induces transcriptional profiles connected to enhanced stress resistance and lifespan. To the best of our knowledge, a connection between beneficial statin effects and JNK-1 and/or DAF-16 in *C. elegans* has not been described to date. However, there are two reports showing that treatment with statins triggers Foxo3a (a mammalian DAF-16 homolog) nuclear translocation and/or an up-regulation of SIRT-1/Foxo3a signaling in mice and human fibroblasts [35, 36]. Like DAF-16, Foxo3a is also known to be involved in the higher probability to survive within the oldest-old cohort [37]. Foxo3a variants were repeatedly shown to be associated with elevated life expectancy in humans [38-40]. Noteworthy, especially the advantage of the G allele of Foxo3 single nucleotide polymorphism (SNP) rs2802292, which is associated with higher Foxo3 activity and longevity in different populations worldwide, was reported to confer a 26 % lower risk for coronary heart disease (CHD)-related death. This advantage is most prominent at older age, which is comparable to the reports on statins [41]. Foxo3a nuclear translocation is inhibited via a kinase cascade stimulated by the insulin receptor IGF1R, a receptor tyrosine kinase. It was shown that a depletion of mevalonic acid reduces the amount of dolichyl phosphate leading to hypoglycosylated Insulin-like growth factor-1 receptor (IGF1R) and thereby less newly synthesized IGF1R at membranes of a human cell [42]. Accordingly, it is feasible that Foxo3a gains higher activity in case of HMG-CoA reductase inhibition at least in part through debilitated insulin receptor abundance. Such an effect could also occur with respect to the *C. elegans* insulin/IGF-1 receptor homolog DAF-2. Based on the amino acid sequence for DAF-2 (isoform a) obtained from wormbase.org (WB WS263) and using NetNGlyc 1.0 Server for prediction of N-glycosylation sites (available from http://www.cbs.dtu.dk/services/NetNGlyc/"), we identified eight highly probable N-glycosylation sites on DAF-2. Correct N-glycosylation of the human insulin receptor is vital for proper signal transduction and localization within the membrane [43]. Furthermore, it was shown that statins are potent inhibitors of insulin- and IGF-mediated proliferation in human adipocyte cell culture [44].

In *C. elegans* limited functionality of DAF-2, as in the *daf-2(e1370)* mutant, results in enhanced lifespan, which depends on DAF-16 activity [45]. However, in *daf-2(e1370)* animals we still see an additional, albeit minor, lifespan extension elicited by lovastatin. This finding suggests that the statin effect observed in *C. elegans* is not or only partially DAF-2 dependent. On the other hand, it indicates that the activity of DAF-16 can still be modulated by statins in this mutant. Indeed, nuclear localization of DAF-16 is about 70 % in a *daf-2(e1370)* population [46]. Hence, the observed elevated expression of *jnk-1* as well as the JNK-1 dependent DAF-16::GFP translocation following statin treatment could still modulate DAF-16 translocation in a *daf-2(e1370)* population. Furthermore, the statin-mediated reduction in aging pigments as well as lifespan extension is fully dependent on JNK-1. Previously, it was shown that DAF-16 activation via JNK-1 causes enhanced thermal stress resistance and lifespan extension in *C. elegans* [47]. Both effects were observed after lovastatin treatment in addition to DAF-16-related differential gene expression. After long-term lovastatin treatment (96 h) we found the longevity-associated small heat-shock protein genes *hsp-16.1* and *hsp-16.2* to be upregulated and a vitellogenin gene (*vit-5*) and a DNAse II homolog (*nuc-1*), which both have lifespan shortening effects, to be downregulated. Thus, we conclude that JNK-1 is the driver for DAF-16 dependent lifespan extension in *C. elegans.*

Taken together, we further unraveled molecular mechanisms underlying cholesterol-independent beneficial effects of statin intake. We showed for the first time that the stress-related kinase JNK-1 and the transcription factor DAF-16/Foxo3a, which are both well conserved up to humans, are necessary for lifespan extension by statins in *C. elegans*.

## Supplementary Materials

The Supplemenantry data can be found online at: www.aginganddisease.org/EN/10.14336/AD.2019.0416.
