# Supplementary material for: Statins Induce a DAF-16/Foxo-dependent Longevity Phenotype via JNK-1 through Mevalonate Depletion in C. elegans
Source: Aging Dis. 2020 Feb 1;11(1):60–72. doi: 10.14336/AD.2019.0416 (PMC6961767; doi:10.14336/AD.2019.0416)
Supplement: Supplementary file 1 — The Supplemenantry data can be found online at: www.aginganddisease.org/EN/10.14336/AD.2019.0416. [file AD-11-1-60-s.pdf]

SUPPLEMENTARY DATA

Statins Induce a DAF-16/Foxo-dependent Longevity Phenotype via JNK-1 through Mevalonate Depletion in *C. elegans*

Andreas Jahn, Bo Scherer, Gerhard Fritz, Sebastian Honnen*

Heinrich Heine University Dusseldorf, Medical Faculty, Institute of Toxicology, D-40225 Dusseldorf, Germany

© 2019. Jahn A et al. Published online at http://www.aginganddisease.org/EN/10.14336/AD.2019.0416

SUPPLEMENTARY DATA

Supplementary Table 1. Mean survival of populations, pre-treated with different statin concentrations, after oxidative or thermal stress.

	Survival ± SD [%]			
Pretreatment/ Posttreatment	**Control**	**50 µM Lovastatin**	**100 µM Lovastatin**	**150 µM Lovastatin**
Wt (adult +1)				
3 h 37 °C	85.62±7.95	89.11±5.73	87.21±3.23	89.31±3.17
4 h 37 °C	64.61±7.05	78.30±3.03*	71.21±6.56	70.00±2.89
5 h 37 °C	45.72±6.12	66.23±6.92*	69.48±2.78*	69.39±3.43*
6 h 37 °C	17.46±9.07	34.12±3.88*	32.83±6.84*	34.21±1.44*
Wt (adult +1)				
4 h 2 mM H_2O_2	57.50±18.87	71.67±20.21	60.42±25.75	63.43±36.12
4 h 4 mM H_2O_2	42.79±22.06	46.39±25.77	26.98±28.85	29.80±22.17
4 h 6 mM H_2O_2	29.74±22.28	32.95±38.51	27.68±33.89	27.78±25.46

**(p≤0.05)*

Each value represents the mean from three independent experiments with at least 20 individuals each (N>60, n=3). *p≤0.05 (1way ANOVA with uncorrected Fisher´s LSD post hoc test).

Supplementary Table 2. Mean chemotactic index (CI) for lovastatin.

**chemotactic index (CI) of lovastatin**
-0.006±0.079

Shown is the mean ± SD from three independent trials (n = 3, N ≥ 300).

© 2019. Jahn A et al. Published online at http://www.aginganddisease.org/EN/10.14336/AD.2019.0416

SUPPLEMENTARY DATA

Supplementary Table 3. Mean or post-reproductive lifespan of each individual trial of populations with different genetic backgrounds with and without statin treatment.

Genetic background(allele) [start of treatment] group	*Mean lifespan ± SEM (days)*			
	**1. trial**	**2. trial**	**3. trial**	**4. trial**
Wt [adult +1]				
control	25,10±1,39	17,08±1,67	23,54±1,27	21,92±0,99
25 µM lovastatin	24,14±1,34	22,18±1,72	24,69±1,34	25,86±0,98*
50 µM lovastatin	23,39±1,72	20,79±1,93	24,76±1,43	26,73±0,98*
100 µM lovastatin	27,13±2,43	25,20±1,66*	24,72±1,47	29,18±1,08*
Wt [adult +8]				
control	11,79±1,22	13,43±0,74	12,74±0,75	
25 µM lovastatin	14,63±1,35	15,47±0,62	15,26±0,76*	
50 µM lovastatin	14,73±1,45	16,65±0,90*	15,78±0,60*	
100 µM lovastatin	15,46±1,87	17,06±0,80*	16,70±0,65*	
Wt [adult+1]				
control	15,42±1,04	18,46±0,91	17,52±1,03	
25 µM simvastatin	16,02±0,83	18,41±1,05	17,94±1,03	
50 µM simvastatin	18,52±1,16*	19,21±1,08	19,78±1,13	
100 µM simvastatin	17,10±1,27	19,97±1,45	20,91±1,21*	
daf-16(mu86)[adult +1]				
control	18,60±0,60	17,38±0,39	15,14±0,69	
100 µM lovastatin	18,74±0,58	17,74±0,33	14,68±0,70	
daf-2(e1370)[adult +1]				
control	22,17±1,27	30,083±1,33	23,93±0,84	
100 µM lovastatin	21,91±1,4	34,96±1,64*	28,37±1,14*	
jnk-1(gk7)[adult +1]				
control	25,39±0,93	19,78±0,47	22,89±0,68	
100 µM lovastatin	23,73±0,96	20,88±0,39	21,09±0,99	

We calculated the mean lifespans within the respective observation period. Usually this is day one of adulthood (adult +1) until death, but for the post-reproductive lifespan (adult +8) it represents only the average number of days between day eight of adulthood and death. The log rank post-hoc test was performed for determining statistical difference in comparison to the control for all treatment groups (*$p \leq 0.05$).

© 2019. Jahn A et al. Published online at http://www.aginganddisease.org/EN/10.14336/AD.2019.0416

SUPPLEMENTARY DATA

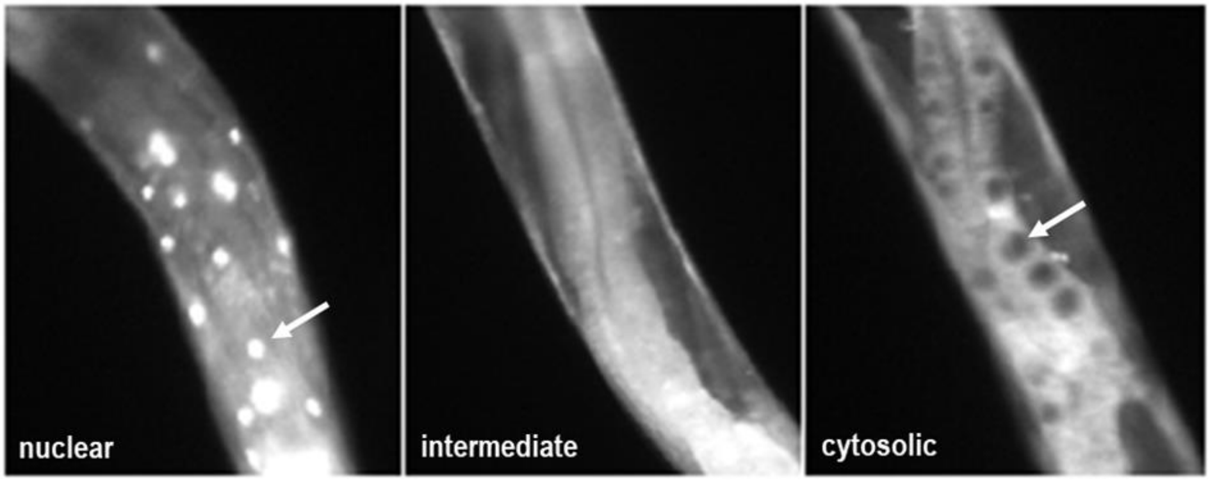

Supplementary Figure 1. Representative images for the three stages of DAF-16::GFP. **(cytosolic)** DAF-16::GFP is only present in the cytosol and not in the nucleus (arrow). **(intermediate)** DAF-16::GFP is localized both in the nucleus and in the cytosol. **(nuclear).** DAF-16::GFP is only present in the nucleus (arrow).

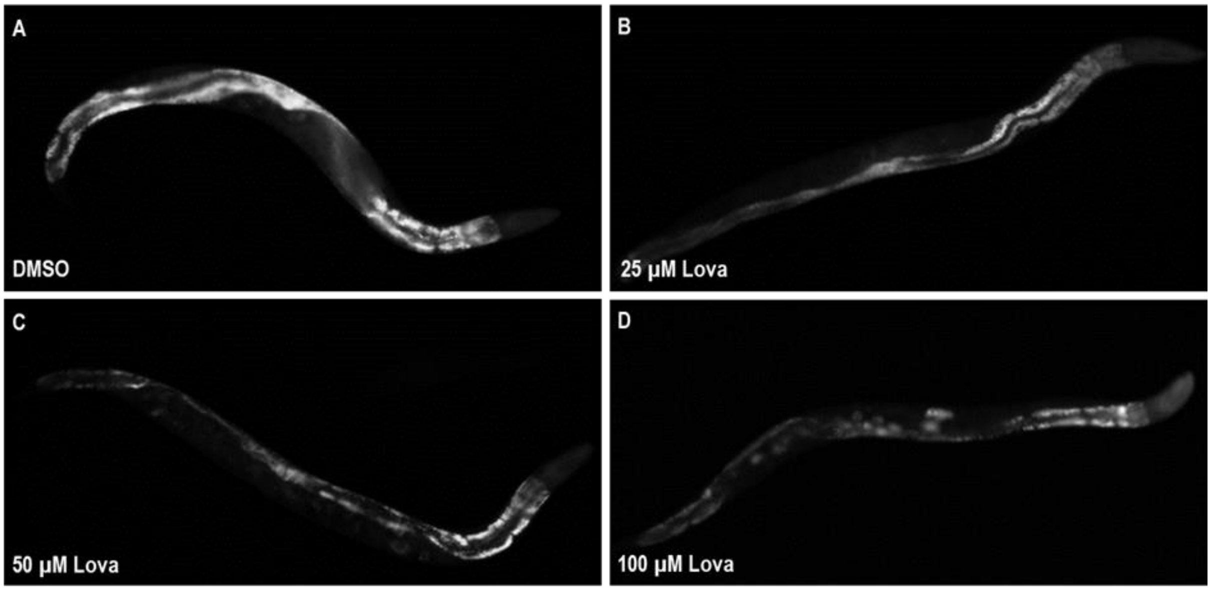

Supplementary Figure 2. Lovastatin reduces the accumulation of aging pigments in the intestine of *C. elegans*. After twelve days of incubation the autofluorescence of aging pigments was determined (DAPI filter: extinction 360-370 nm; emission 420-460 nm). Treatment with 100 µM lovastatin (D) reduces accumulation of aging pigments (about 40 %) compared to the control group (A). Shown are representative images of the accumulation of aging pigments of at least 3 individual trials (n = 3, N ≥ 30).

© 2019. Jahn A et al. Published online at http://www.aginganddisease.org/EN/10.14336/AD.2019.0416

SUPPLEMENTARY DATA

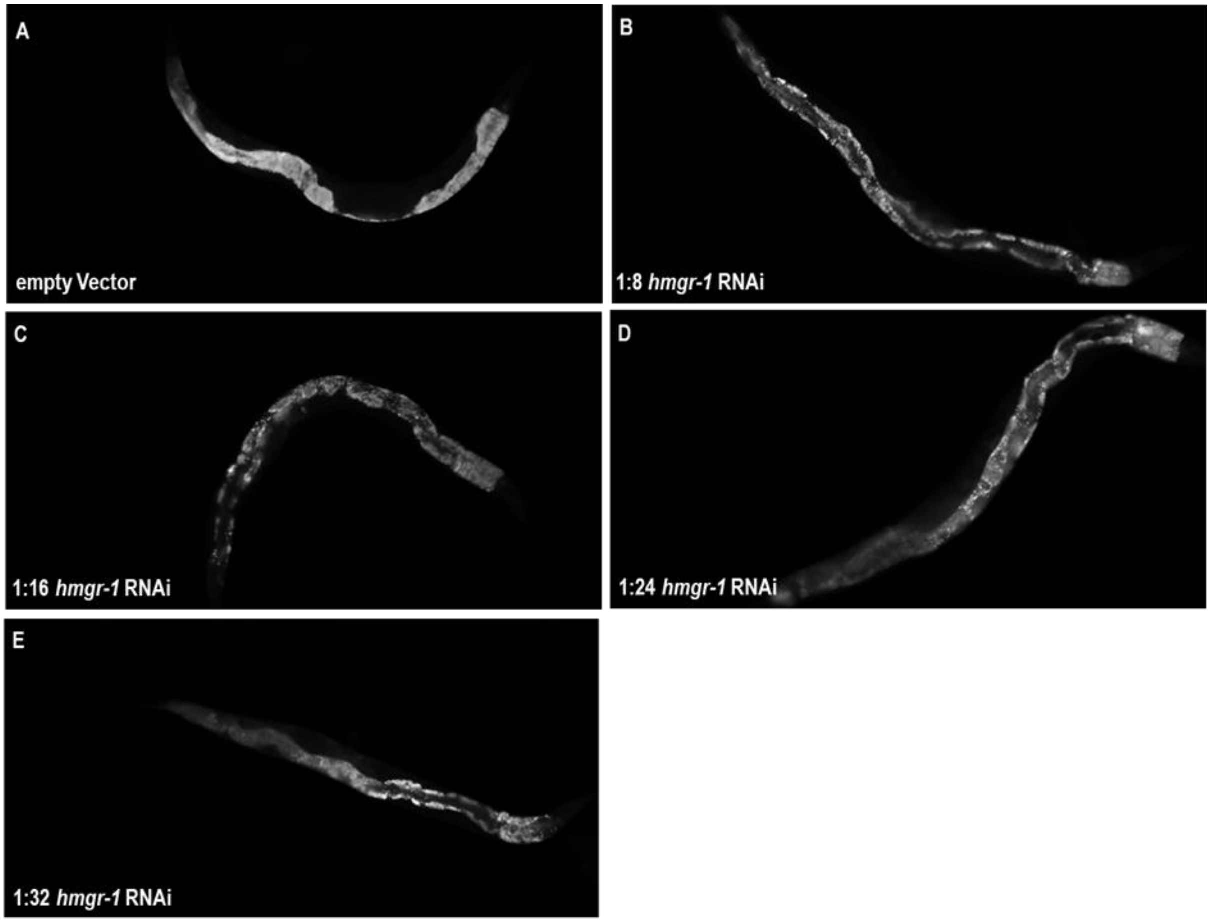

Supplementary Figure 3. Slight knockdown of HMG-CoA-reductase reduces the accumulation of age pigments in the intestine of *C. elegans*. After twelve days of incubation the autofluorescence of aging pigments was determined (DAPI filter: extinction 360-370 nm; emission 420-460 nm). Slight knockdown of HMG-CoA-reductase mRNA by RNAi (B-E) reduces accumulation of aging pigments (about 40 %) compared to the control group (A). Shown are representative images of the accumulation of aging pigment of at least 3 individual trials (n = 3, N ≥ 40).

© 2019. Jahn A et al. Published online at http://www.aginganddisease.org/EN/10.14336/AD.2019.0416

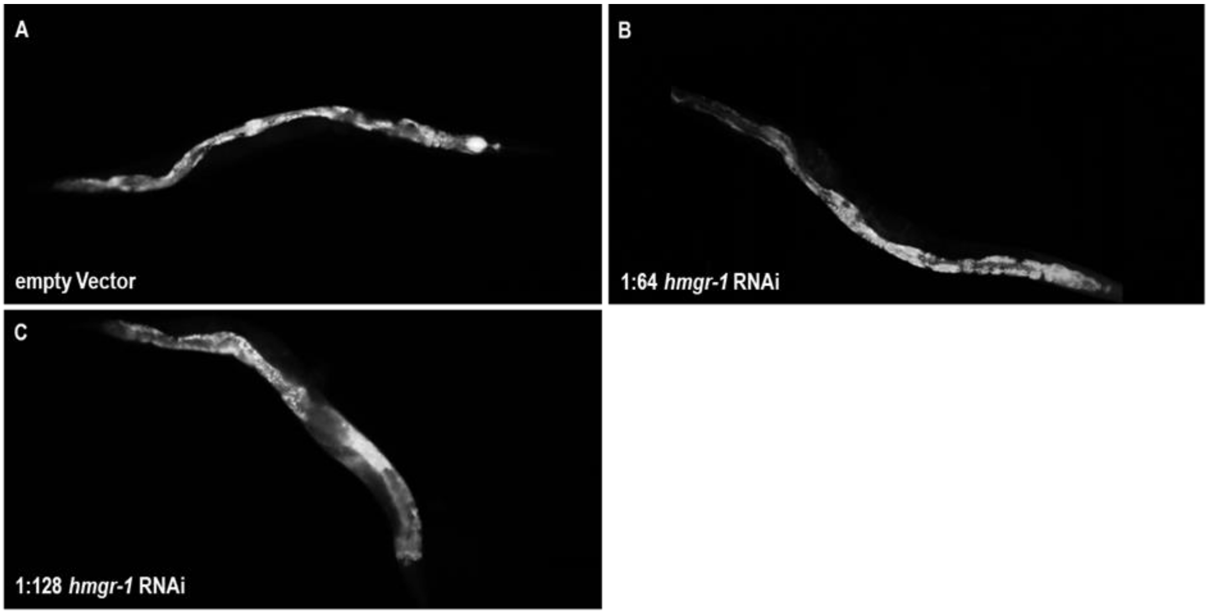

Supplementary Figure 4. Very high dilutions of *hmgr-1(RNAi)* bacteria fail to reduce the accumulation of age pigments. After twelve days of incubation the autofluorescence of aging pigments was determined (DAPI filter: extinction 360-370 nm; emission 420-460 nm). In case of very high dilutions of *hmgr-1(RNAi)* bacteria (B and C) there is no effect on accumulation of aging pigments in *C. elegans* (A). Shown are representative images of the accumulation of aging pigment of at least 3 individual trials (n = 3, N ≥ 30).

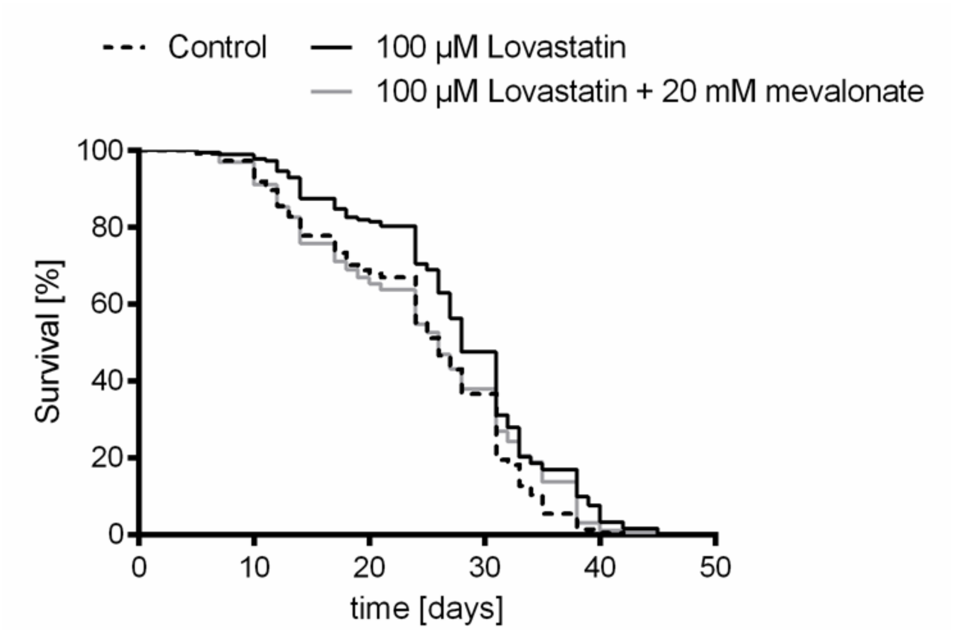

Supplementary Figure 5. Mevalonate reverts the lovastatin-induced longevity in *C. elegans*. Synchronized nematode populations (L4) were subdivided to DMSO (control), 100 µM lovastatin or 100 µM lovastatin + 20 mM mevalonate. Shown are Kaplan-Meier survival plots for the time of treatment. Supplementation with 20 mM mevalonate suppresses the lifespan extension by 100 µM lovastatin. The log rank post-hoc test showed statistical difference of the 100 µM lovastatin group in comparison to "control" as well as to "100 µM lovastatin + 20 mM mevalonate" (n = 3, N ≥ 90; *p≤0.05).

© 2019. Jahn A et al. Published online at http://www.aginganddisease.org/EN/10.14336/AD.2019.0416

SUPPLEMENTARY DATA

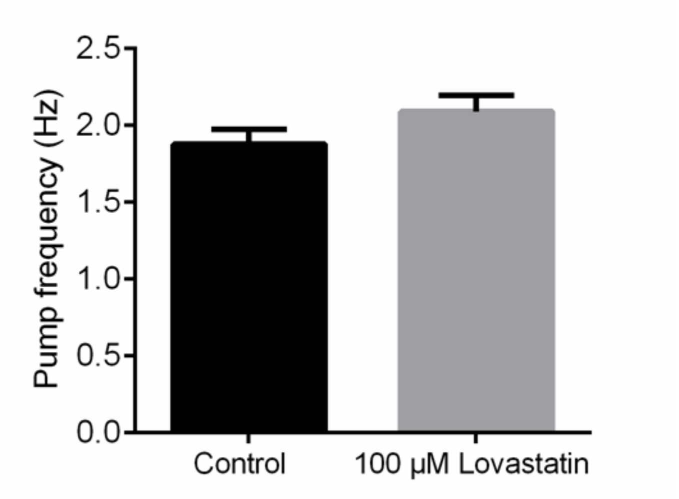

Supplementary Figure 6. Pump frequency of wild type *C. elegans* populations after 24 h treatment with 100 μM lovastatin or 0.2 % DMSO (Control). Shown is the mean ± SD from three independent trials (n = 3, N ≥ 30). There is no statistically significant difference in the pump frequency of the two groups. Test for statistical difference was performed using unpaired Student's t-test.

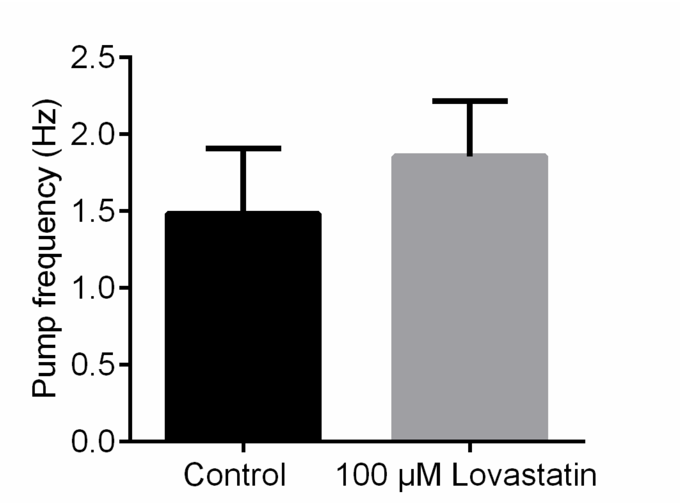

Supplementary Figure 7. Pump frequency of wild type *C. elegans* in the presence of 100 μM lovastatin or 0.2 % DMSO (Control). Shown is the mean ± SD from three independent trials (n = 3, N ≥ 30). There is no statistically significant difference in the pump frequency of the two groups. Test for statistical difference was performed using Student's t-test.

© 2019. Jahn A et al. Published online at http://www.aginganddisease.org/EN/10.14336/AD.2019.0416

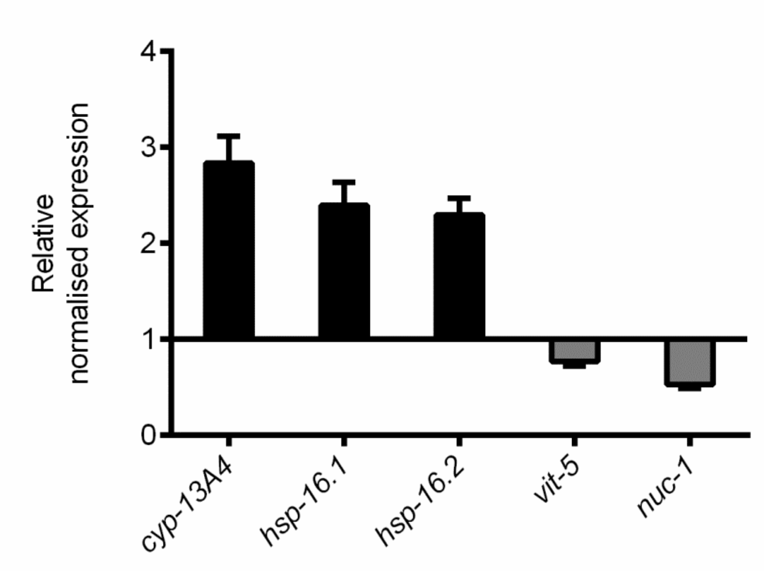

Supplementary Figure 8. Expression levels of certain DAF-16 target genes are modulated after 96 h incubation with lovastatin. Expression levels of different DAF-16 target genes were investigated in wild type *C. elegans* populations after 96 h treatment with 100 µM lovastatin or 0.2 % DMSO. Shown are the expression changes for *cyp-14A4, hsp-16.1, hsp-16.2, vit-5 und nuc1* mRNA. The expression of the target genes which are activated by DAF-16 are also upregulated after lovastatin treatment. The opposite is true for genes that are downregulated by DAF-16. Shown is the mean ± SEM from three technical replicates (cDNA form about 2000 animals).

© 2019. Jahn A et al. Published online at http://www.aginganddisease.org/EN/10.14336/AD.2019.0416

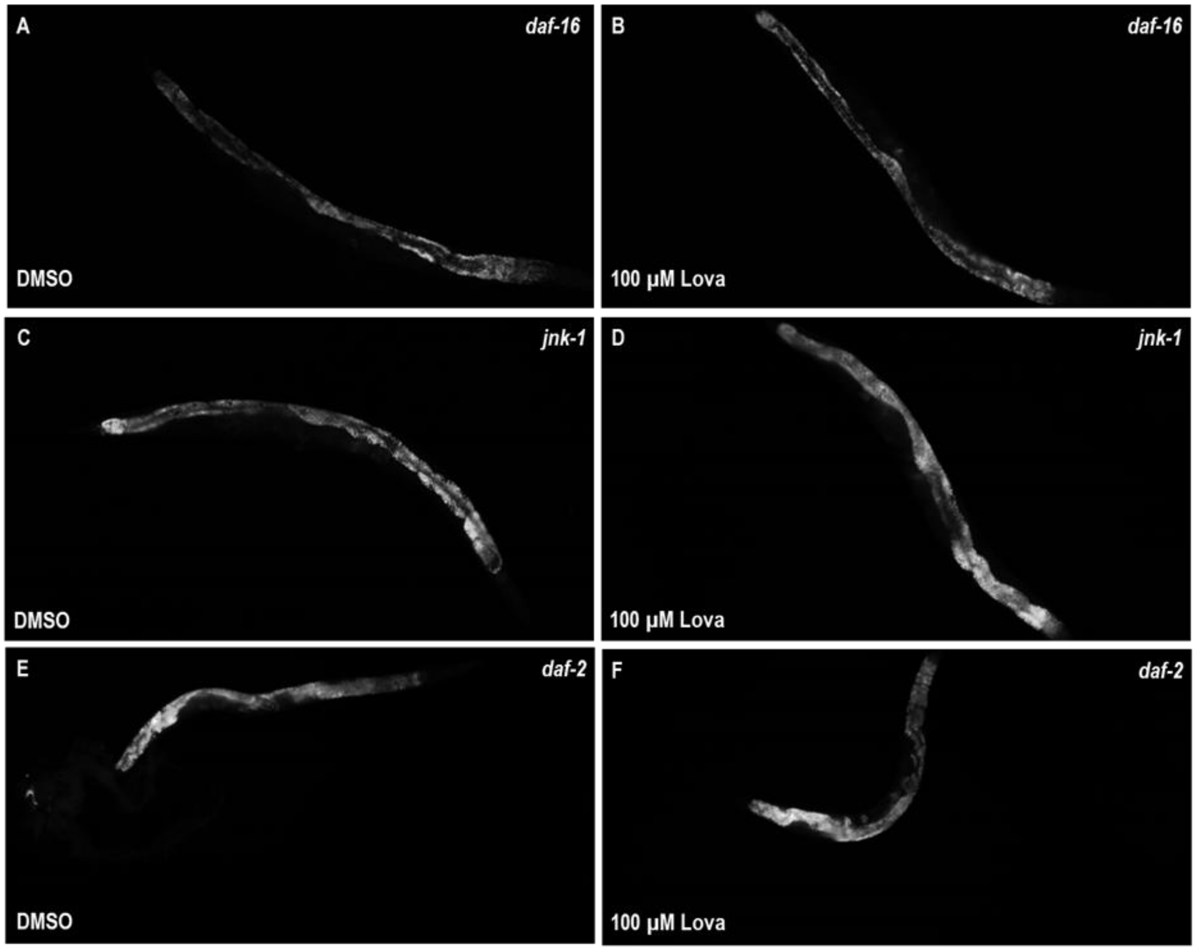

Supplementary Figure 9. Without DAF-16, JNK-1 or DAF-2 lovastatin has no effect on the accumulation of age pigments. After twelve days of incubation the autofluorescence of aging pigments was determined (DAPI filter: extinction 360-370 nm; emission 420-460 nm). Shown are representative images of the accumulation of aging pigment of at least three individual trials ($n = 3$, $N \geq 30$).

© 2019. Jahn A et al. Published online at http://www.aginganddisease.org/EN/10.14336/AD.2019.0416
